# The influence of temperature on mortality and its Lag effect: a study in four Chinese cities with different latitudes

**DOI:** 10.1186/s12889-016-3031-z

**Published:** 2016-05-04

**Authors:** Junzhe Bao, Zhenkun Wang, Chuanhua Yu, Xudong Li

**Affiliations:** 1 Department of Epidemiology and Biostatistics, School of Public Health, Wuhan University, #185 Donghu Road, Wuhan, 430071 China; 2 Global Health Institute, Wuhan University, #8 Donghunan Road, Wuhan, 430072 China; 3 Office of Epidemiology, Chinese Center for Disease Control and Prevention, #155 Changbai Road, Changping District, Beijing, 102206 China

**Keywords:** Temperature–mortality relationship, Latitude, China, Lag effect

## Abstract

**Background:**

Global climate change is one of the most serious environmental issues faced by humanity, and the resultant change in frequency and intensity of heat waves and cold spells could increase mortality. The influence of temperature on human health could be immediate or delayed. Latitude, relative humidity, and air pollution may influence the temperature–mortality relationship. We studied the influence of temperature on mortality and its lag effect in four Chinese cities with a range of latitudes over 2008–2011, adjusting for relative humidity and air pollution.

**Methods:**

We recorded the city-specific distributions of temperature and mortality by month and adopted a Poisson regression model combined with a distributed lag nonlinear model to investigate the lag effect of temperature on mortality.

**Results:**

We found that the coldest months in the study area are December through March and the hottest months are June through September. The ratios of deaths during cold months to hot months were 1.43, 1.54, 1.37 and 1.12 for the cities of Wuhan, Changsha, Guilin and Haikou, respectively. The effects of extremely high temperatures generally persisted for 3 days, whereas the risk of extremely low temperatures could persist for 21 days. Compared with the optimum temperature of each city, at a lag of 21 days, the relative risks (95 % confidence interval) of extreme cold temperatures were 4.78 (3.63, 6.29), 2.38 (1.35, 4.19), 2.62 (1.15, 5.95) and 2.62 (1.44, 4.79) for Wuhan, Changsha, Guilin and Haikou, respectively. The respective risks were 1.35 (1.18, 1.55), 1.19 (0.96, 1.48), 1.22 (0.82, 1.82) and 2.47 (1.61, 3.78) for extreme hot temperatures, at a lag of 3 days.

**Conclusions:**

Temperature–mortality relationships vary among cities at different latitudes. Local governments should establish regional prevention and protection measures to more effectively confront and adapt to local climate change. The effects of hot temperatures predominantly occur over the short term, whereas those of cold temperatures can persist for an extended number of days.

**Electronic supplementary material:**

The online version of this article (doi:10.1186/s12889-016-3031-z) contains supplementary material, which is available to authorized users.

## Background

Global climate change is one of the most serious environmental issues confronting humanity. The Intergovernmental Panel on Climate Change (IPCC) reported that average temperature data showed a global warming trend of 0.85 (0.65 to 1.06) °C from 1880 to 2012. The IPCC projected that there would be an increase in the duration, intensity and spatial extent of heat waves and warm spells during the next decade [1]. Many studies have found that heat waves and cold spells could lead to an increase in the number of temperature-related deaths [2, 3]. Temperature–morality curves have been described as U-, V-, or W-shaped, with U- or V-shaped representing the peaks of deaths occurring at very high and low temperatures; the U-shaped curve has a more pronounced decrease and increase in mortality with a rise in temperature than the V-shaped curve. The W-shaped curve represents the fact that increased mortality levels are distributed at high and low temperatures. There is a small peak of deaths between high and low temperatures, which may result from the response to a rise in temperature. People may ignore measures necessary to adapt to temperature change, resulting in a slight increase in mortality at the beginning, which then decreases after some adaptation measures are taken [4–8]. Previous studies have found that the adverse effects of temperatures on human health not only occur in the short term but also after a delay [9, 10]. A distributed lag nonlinear model (DLNM) has been developed to study these delayed effects and the nonlinear exposure–response relationship simultaneously [11].

The influence of temperature on human health varies among countries and regions [12]. For more effective adaptation to climate change, governments should establish regional prevention and control systems based on the corresponding influences of temperature on human health. Many studies have shown that latitude exerts an important influence on the relationship between temperature and mortality. Generally, people living at low latitudes have a relatively weak adaptive capacity to cold and a strong adaptive capacity to heat, and the reverse has also been shown [5, 13]. Additionally, humidity [14, 15] and air pollution [16, 17] have been reported to have an effect on mortality rates.

We studied the relationship between temperature and mortality in four Chinese cities with different latitudes, and adjusted for the possible influence of relative humidity and air pollution. We used a quasi-Poisson analysis to investigate the relationship between temperature and mortality and a DLNM to study the delayed effect of temperature on mortality. Our findings will provide useful information for understanding the health effects of temperature at different latitudes in China.

## Methods

### Study sites

**Fig. 1:**
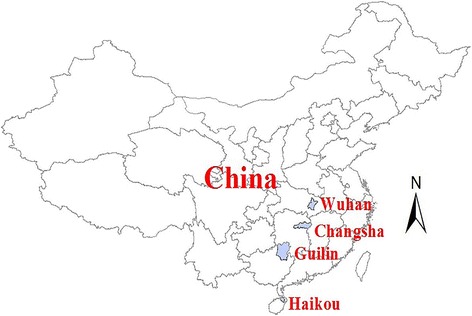
Locations of studied cities

We studied four cities with different latitudes (Wuhan, Changsha, Guilin, and Haikou, as shown in Fig. 1). These cities are the provincial capitals of their respective provinces in China, except for Guilin, which is a famous tourist destination in Guangxi. Wuhan has a subtropical monsoon humid climate; the city is located where the world’s third longest river, the Yangtze, joins its largest tributary, the Han River. Wuhan is an important transportation hub and a scientific research and education center. The city has four distinct seasons, with cold and wet winters and hot, humid summers. Changsha has a subtropical monsoon climate, with four distinct seasons including cold winters and hot summers; it is a major and highly industrialized city in China. The climate in Guilin also has subtropical monsoons but is relatively mild, with neither extreme cold in winter nor excessive heat in summers. Haikou has a tropical marine climate, with hot and rainy summers and mild winters.

Owing to data availability, we were only able to use data from two districts in Wuhan (Qiaokou and Jiangan) and one district each in Changsha (Tianxin), Guilin (Xiufeng), and Haikou (Meilan); according to the Sixth National Population Census of China, in 2010 the populations in these four districts were 1.73 million, 0.47 million, 0.16 million and 0.62 million, respectively. The regional gross domestic product was 551.58, 454.70, 110.86 and 59.06 billion CNY, respectively, and per capita annual disposable income was 20.81, 22.81, 17.95 and 16.72 thousand CNY, respectively [18].

### Data collection

Meteorological data of the four cities for 2008–2011, including daily mean temperature, minimum temperature, maximum temperature, and relative humidity, were obtained from the China Meteorological Data Sharing Service System [19].

Daily mortality data of the four cities were collected from the Provincial Centers for Disease Control and Prevention. Causes of death were encoded according to the 10th International Classification of Diseases (ICD-10). The term “non-accidental death” referred to all causes of death, excluding external causes such as injury and poisoning (ICD-10 codes: A00-R99). The Air Pollution Index (API) is an indicator of the ambient air quality, which is calculated using the measured concentrations of sulfur dioxide (SO_2_), nitrogen dioxide (NO_2_) and respirable particulate matter (PM_10_), according to the methodology of the World Health Organization and European Centre for Environment and Health, at all monitoring stations throughout each city-specific territory. A lower API indicates better air quality [20]. API data were collected from the Ministry of Environmental Protection of the People’s Republic of China.

### Data analysis

Relationships of mortality and certain risk factors are typically investigated using Poisson regression [21, 22]. However, some researchers have hypothesized that quasi-Poisson analysis should be used if overdispersion is found with Poisson regression, and that the R qcc package provides a test for this phenomenon [23, 24]. We used a DLNM to assess the effects of daily temperature on mortality with different lag days [10, 11]. To explore the effects of temperature on daily mortality, factors such as long-term and seasonal trends, day of the week (DOW), relative humidity (RH), and API were introduced into the model as covariates.

$$ \mathrm{LogE}\left[{Y}_t\right]=\upalpha + \upbeta {T}_{t,l}+NS\left(R{H}_t,\ df=3\right)+NS\left( Time,\ df=7*4\right)+NS\left( Season,\ df=3\right)+NS\left(AP{I}_t,df=3\right)+ DO{W}_t $$

Describes the model we selected, where *E*[*Y*_*t*_] denotes the expected number of nonaccidental deaths on day *t*; α is the intercept; *T*_*t,l*_ is a matrix obtained by applying the temperatures, with *l* referring to the lag days and *β* the coefficient. *NS* represents the natural spline function; *RH*_*t*_ is the daily relative humidity on day *t*; the degrees of freedom (df) is 3, and the df of each variant is confirmed by the Akaike information criterion for the quasi-Poisson models [25]. *Time* represents the long-term temporal trend; *Season* represents the seasonal trend; *API*_*t*_ is the air pollution index on day t; *DOW*_*t*_ is the day of the week on day *t*.

We used lag-stratified natural cubic spline models to explore the nonlinear and delayed temperature–mortality association. We adopted city-specific optimum temperature as the reference value, which was determined by comparing the relative risks (RR) of temperatures at 3-day lag, and the temperature with the lowest RR was set as the optimum temperature. A natural cubic spline with 4 df was applied to the daily mean temperature. Knots of the mean temperatures were placed at equally spaced quantiles. Lag stratification was defined as 5 df for the mean temperature. The knots of the lag calculation were set at equally spaced values on the log scale of the lags. We plotted RR against the temperature and lags to show the entire relationship between mean temperature and mortality. Extreme cold temperatures were set as the first percentile of the daily mean temperature, and the lag days were set to 21. Extreme hot temperatures were set as the 99th percentile of daily mean temperature, and the lag days were set to 3.

The sensitivity analyses were performed by changing the df of long-term trends and API, with df (time) = 5, 6, 7 or 8/year, and df (API) = 2, 3, 4, or 5. We used R 3.0.2, software for the analyses. The distributed lag nonlinear models were specified with the “dlnm” package, and tests for over dispersion were performed using the “qcc” package.

## Results

Table 1 shows the city-specific distribution of temperature, relative humidity, API, and daily mortality counts. The temperature of the four cities showed an increasing trend from north to south, whereas the API had a decreasing trend. The relative humidity of Wuhan and Haikou were higher than those of the other two cities.

**Table 1:** Summary statistics of weather conditions, air pollution, and daily mortality for four cities in China during 2008–2011

Variables	City	Mean (STD^a^)	Median (IQR^b^)	Min	Max
Temperature (°C)	Wuhan	17.15(9.55)	18.1(16.1)	–2.7	35.3
	Changsha	18.27(9.35)	19.2(16.2)	–3	35.5
	Guilin	19.57(8.17)	21.3(13.8)	–0.7	32.6
	Haikou	23.90(4.76)	25.4(6.7)	8.7	31.6
Relative humidity (%)	Wuhan	73.84(12.96)	75.0(17.0)	21.0	100.0
	Changsha	72.95(13.06)	74.0(19.0)	28.0	96.0
	Guilin	70.83(14.33)	71.0(19.0)	22.0	100.0
	Haikou	80.54(8.01)	81.0(10.0)	47.0	98.0
Air Pollution Index	Wuhan	77.77(33.64)	74.0(38.0)	14.0	500.0
	Changsha	68.66(27.09)	67.0(29.0)	11.0	443.0
	Guilin	51.77(19.44)	51.0(26.0)	15.0	179.0
	Haikou	38.48(14.21)	35.0(21.0)	6.0	99.0
Daily non-accidental deaths	Wuhan	20.94(7.74)	21.0(10.0)	1.0	49.0
	Changsha	3.34(1.98)	3.0(2.0)	0.0	11.0
	Guilin	1.16(1.13)	1.0(2.0)	0.0	6.0
	Haikou	2.13(1.91)	2.0(2.0)	0.0	15.0

^a^Standard deviation

^b^Interquartile range

**Fig. 2:**
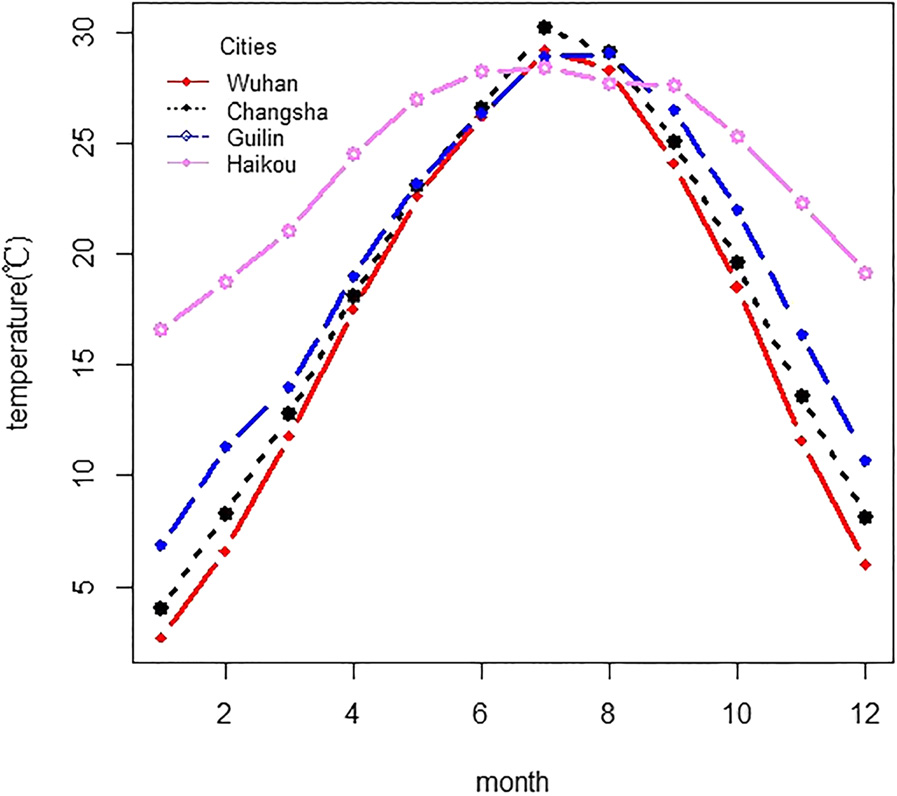
City-specific distributions of daily mean temperature, by month

**Fig. 3:**
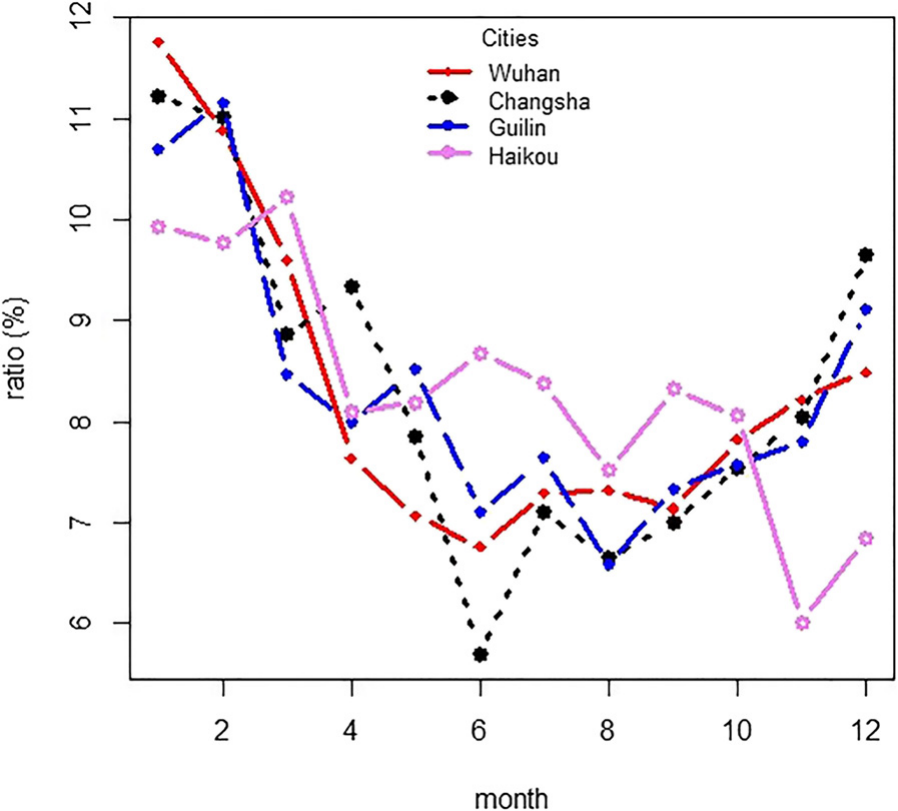
City-specific distribution of ratios of deaths by month.^*^^*^ Ratios of deaths calculated using number of deaths during an entire year as denominator and number of deaths during a specific month as numerator

Figure 2 shows the city-specific distribution of daily mean temperature by month. We found that the primary cold months were December, January, February and March, and the hot months were June through September. Figure 3 shows that most deaths occurred in the cold months, and ratios of deaths in the cold months to hot months were 1.43, 1.54, 1.37 and 1.12 for Wuhan, Changsha, Guilin and Haikou, respectively. The risk ratios were higher in Wuhan and Changsha than in Guilin and Haikou.

**Fig. 4:**
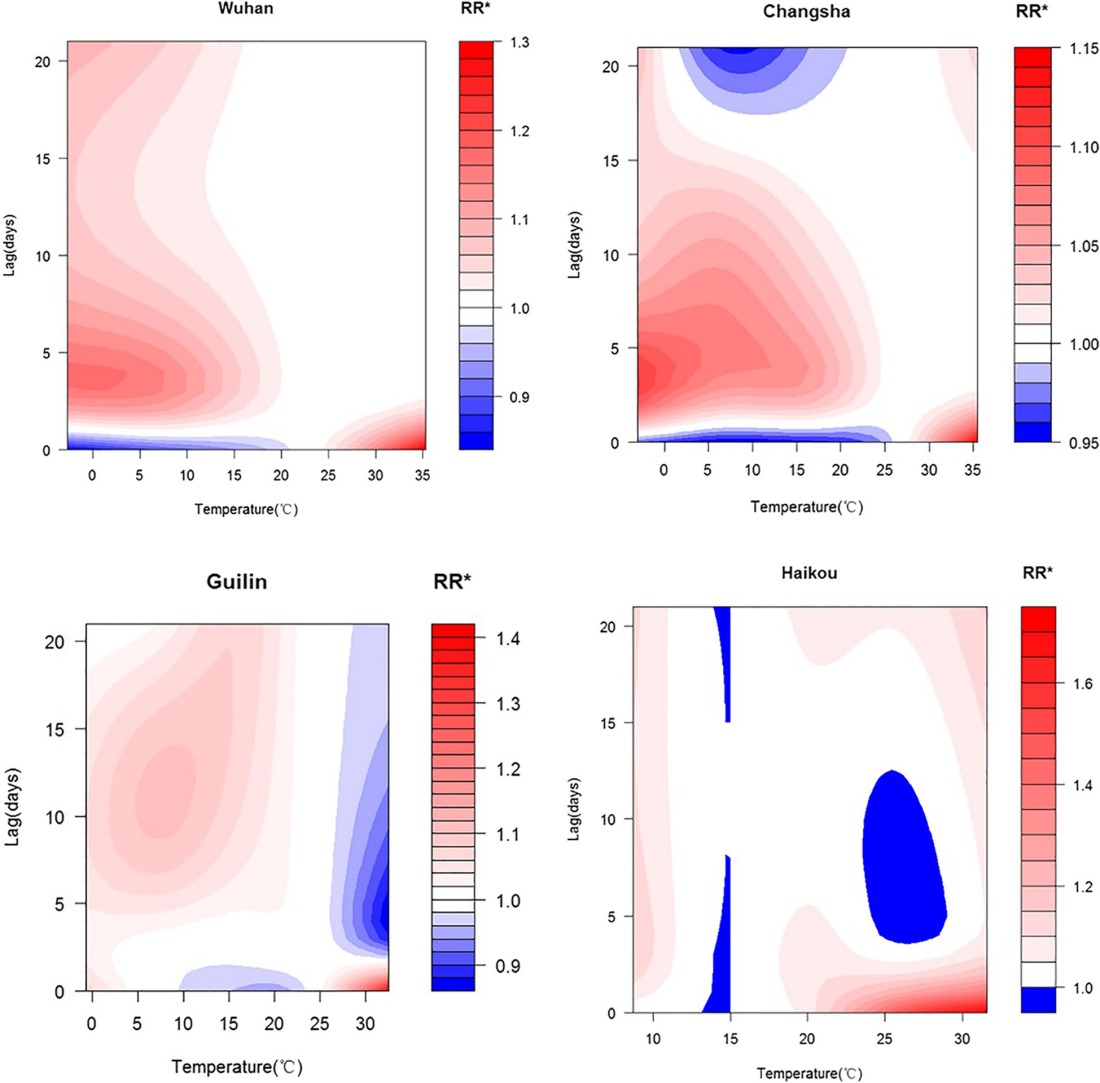
Contour plots of temperature–mortality relationships in the four cities

Contour plots (Fig. 4) show the relationships between mean temperature and mortality at a lag of 21 days. For extremely high temperatures, the effect was the strongest at lag0 and then declined rapidly. Its effect was generally retained for three days at Wuhan, Changsha and Guilin, whereas for Haikou, the effect of hot temperatures could be maintained longer. For the extremely low temperatures, the risk was the smallest at lag0, followed by an incremental increase, except at Guilin, where the low temperature remained at a relatively low risk level within a lag of 21 days.

**Fig. 5:**
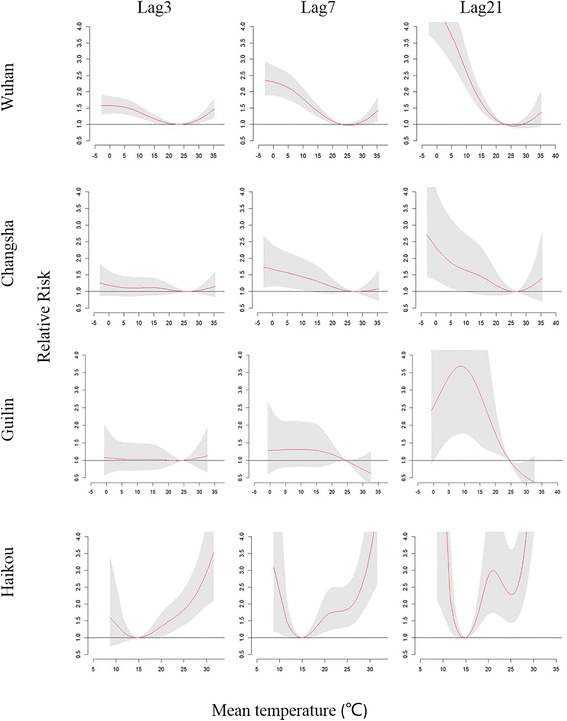
Estimated effects of temperature on mortality at various lag times.^*^^*^ Red lines represent relative risks and gray regions indicate 95 % confidence intervals

We drew city-specific temperature–mortality curves for different lag times (Fig. 5). The curves were similarly V-shaped at a lag of 3 days, which may indicate the short-term effects of temperature on mortality. Then, with increasing lag time, the risk of cold temperatures increased gradually, whereas the increase in risk of hot temperatures was much smaller. Table 2 shows the RR of extreme cold and hot temperatures compared with the optimum temperatures, which were 22.9, 26.9, 24.5 and 15.0 °C for Wuhan, Changsha, Guilin and Haikou. RRs (95 % confidence interval) of the extreme cold temperatures were 4.78 (3.63, 6.29), 2.38 (1.35, 4.19), 2.62 (1.15, 5.95) and 2.62 (1.44, 4.79), respectively, at a lag of 21 days. RRs of the extreme hot temperatures were 1.35 (1.18, 1.55), 1.19 (0.96, 1.48), 1.22 (0.82, 1.82) and 2.47 (1.61, 3.78), respectively, at a lag of 3 days.

**Table 2:** Relative risk (95 % confidence interval) of extreme cold and hot temperatures on mortality, compared with city-specific optimum temperatures of the four cities^*^

Cities	Extreme cold	Extreme hot
Wuhan	4.78 (3.63,6.29)	1.35 (1.18,1.55)
Changsha	2.38 (1.35,4.19)	1.19 (0.96,1.48)
Guilin	2.62 (1.15,5.95)	1.22 (0.82,1.82)
Haikou	2.62 (1.44,4.79)	2.47 (1.61,3.78)

^*^Extreme cold temperatures were set as the first percentile of daily mean temperature and lag period was set to 21 days. Extreme hot temperatures were set as 99th percentile of daily mean temperature and lag period was set to 3 days

Sensitivity analyses showed that with changing the df of time trends and API, the extreme cold and hot effects were robust to alternative models (Additional file 1: Table S1).

## Discussion

We found that mortality was higher in the cold months than in the hot months, and that the seasonal pattern was lower for mortality in the southern cities in China than in the central cities. This finding concurs with a number of previous studies [26–29]. The seasonality of mortality rates might be attributed to the following reasons: exposure to cold increases energy expenditure, peripheral vasoconstriction and cardiac afterload, which can trigger myocardial ischemia and stroke [26, 30]; influenza is more prevalent in winter [31]; and differences in lifestyle factors such as dietary habits [32] and physical activity [33] between cold and hot months. Considering global climate change, some researchers have suggested that an increase of heat-related mortality would be somewhat offset by reductions in cold-related mortality, and that even overall mortality might decrease for a certain period [34, 35], but other researchers have disagreed [36, 37]. It is possible that in the future, mortality might be reduced with winter-dominant warming and increased with pronounced summer warming [35].

We found that high and low temperatures were associated with increased mortality in the four studied cities, and the effect of high temperatures on mortality was predominantly within 3 days. The effect of low temperatures could persist for 21 days. This finding is consistent with some studies [10, 38]. For Haikou, the effect of high temperatures could last for an extended period, possibly because the temperature is persistently high in summer. We only examined a small sample, and this phenomenon requires further research.

We found that at a lag of 3 days, which reflects short-term effects of the temperature on mortality rate, the curve of their relationship had a V shape. As lag time increased, the shape of the curves changed, and RRs associated with low temperatures increased gradually, whereas the risks associated with high temperatures did not substantially increase but actually decreased. At a lag of 21 days, the temperature–mortality curve appeared to be U-shaped for Wuhan and Changsha. For Haikou, the curve appeared to have a W shape, which is consistent with a previous study in China by Jinan [7]. For Guilin, the mortality risk decreased with temperature after a lag of 21 days, whereas the curve was V-shaped at a lag of 3 days. This might be because summer temperatures are not excessively high in Guilin; the effect of high temperatures typically persists for 3 days and the mortality effect of those temperatures after that is weak, so the effect estimated by the model may not have been exact. A similar phenomenon was found in Charlotte, a city in the southeastern United States of America [5]. The study sample in Guilin was relatively small, so the generalizability of this result is limited. Because of the long lag effect of cold temperatures and relatively short lag effect of hot temperatures, we believed that the effect of extreme cold temperatures could be calculated at a lag of 21 days, whereas the effect of extreme hot temperatures could be calculated at a lag of 3 days. This method was also used in a previous study [39].

The estimated effects of extreme cold and hot temperatures on mortality varied by city. This might be because the four cities have different temperature distributions (Fig. 2). Generally, the cumulative effects of extreme cold were stronger than extreme heat, in agreement with the seasonal pattern discussed above. This phenomenon was also found in a previous study in China [10]. The greatest effect of extreme cold was in Wuhan, where the mean temperature was the coldest of the four cities. The weakest effect was in Haikou, where both the mean and minimum temperatures were the highest of the four cities. The greatest effect of extreme heat was also in Haikou.

Our study has the following limitations. First, because of the limited availability of data, we only included cities in central and southern China, ignoring northern cities. We will try to correct this limitation in a future study. Second, samples were confined to one or two districts in the studied cities, restricting the generalizability of our results. Finally, socio-demographic factors, including age, sex, and income, were not considered. Those factors may influence the temperature–mortality relationship.

## Conclusions

More deaths occurred during cold months in the studied cities, and we found that cities at different latitudes have diverse temperature–mortality relationships. The effects of hot temperatures are predominantly sudden, whereas those of cold temperatures can persist for an extended period. Local governments should establish regional prevention and protection measures to more effectively confront and adapt to climate change.

### Ethics approval and consent to participate

Ethical approval was given by the medical ethics committee of Wuhan University School of Medicine with the following reference number: YXBGW0016.

### Consent for publication

The mortality data used in this study is the number of deaths per day, and our paper does not contain any individual personal’s data in any form.

### Availability of data and materials

Because that we have signed nondisclosure agreements with Chinese Center for Disease Control and Prevention, so the data used in this paper will not be shared.

## Additional files

Additional file 1: Table S1. Sensitivity of extreme cold and hot effects on mortality to change in degrees of freedom used to model time and API splines (Wuhan). (DOC 53 kb)

## Abbreviations

API: air pollution index
df: degrees of freedom
DLNM: distributed lag non-linear model
DOW: day of the week
ICD-10: 10th International Classification of Diseases
IQR: Interquartile range
NS: natural cubic spline
RH: relative humidity
RR: relative risk
STD: standard deviation
