# Supplementary material for: The influence of temperature on mortality and its Lag effect: a study in four Chinese cities with different latitudes
Source: BMC Public Health. 2016 May 4;16:375. doi: 10.1186/s12889-016-3031-z (PMC4855424; doi:10.1186/s12889-016-3031-z)
Supplement: Supplementary file 1 — Sensitivity of extreme cold and hot effects on mortality to change in degrees of freedom used to model time and API splines (Wuhan). (DOC 53 kb) [file 12889_2016_3031_MOESM1_ESM.doc]

| Table S1. Sensitivity of extreme cold and hot effects on mortality to change in degrees of freedom used to model time and API splines (Wuhan). | | | | | | |
| --- | --- | --- | --- | --- | --- | --- |
| Df(time/year) | Df(API) | Extreme cold | |  | Extreme hot | |
|  |  | RR | 95%CI |  | RR | 95%CI |
| 5 | 2 | 4.74 | 3.60-6.25 |  | 1.35 | 1.18-1.54 |
|  | 3 | 4.80 | 3.64-6.31 |  | 1.38 | 1.21-1.57 |
|  | 4 | 4.80 | 3.64-6.32 |  | 1.38 | 1.20-1.57 |
|  | 5 | 4.80 | 3.64-6.32 |  | 1.38 | 1.20-1.57 |
| 6 | 2 | 4.73 | 3.58-6.23 |  | 1.35 | 1.18-1.55 |
|  | 3 | 4.78 | 3.63-6.29 |  | 1.38 | 1.20-1.58 |
|  | 4 | 4.78 | 3.63-6.30 |  | 1.38 | 1.20-1.58 |
|  | 5 | 4.78 | 3.63-6.30 |  | 1.38 | 1.20-1.58 |
| 7 | 2 | 4.74 | 3.59-6.25 |  | 1.36 | 1.18-1.55 |
|  | 3 | 4.79 | 3.63-6.31 |  | 1.38 | 1.21-1.58 |
|  | 4 | 4.79 | 3.63-6.31 |  | 1.38 | 1.21-1.58 |
|  | 5 | 4.79 | 3.63-6.31 |  | 1.38 | 1.20-1.58 |
| 8 | 2 | 4.72 | 3.58-6.22 |  | 1.36 | 1.19-1.56 |
|  | 3 | 4.77 | 3.62-6.29 |  | 1.39 | 1.21-1.60 |
|  | 4 | 4.77 | 3.62-6.29 |  | 1.39 | 1.21-1.60 |
|  | 5 | 4.77 | 3.62-6.29 |  | 1.39 | 1.21-1.59 |
